# Dietary Antioxidant and Flavonoid Intakes Are Reduced in the Elderly

**DOI:** 10.1155/2015/843173

**Published:** 2015-07-08

**Authors:** Małgorzata Elżbieta Zujko, Anna Maria Witkowska, Anna Waśkiewicz, Iwona Mirończuk-Chodakowska

**Affiliations:** ^1^Department of Food Commodities Science and Technology, Medical University, Szpitalna 37, 15-295 Bialystok, Poland; ^2^Department of Epidemiology, Cardiovascular Disease Prevention and Health Promotion, Institute of Cardiology, Niemodlińska 33, 04-635 Warsaw, Poland

## Abstract

The objective of this study was to determine sources and patterns of antioxidant and flavonoid intakes in the elderly (61–74 yrs) in comparison with young (20–40 yrs) and middle age (41–60 yrs) groups in a cross-sectional study. More than 6000 subjects of both genders, aged 20–74 years, participants of the National Multicenter Health Survey (WOBASZ) took part in this study. Daily food consumption was estimated by the single 24-hour dietary recall. Dietary total antioxidant capacity (TAC) and flavonoid content (FC) were calculated according to the amount of food consumed by the participants combined with antioxidant capacity and flavonoid contents in foods. Food consumption, dietary TAC, and FC were significantly lower in the elderly, especially elderly women in comparison to the young and middle age groups. The consumption of tea, coffee, and apples was associated with the largest contribution to dietary TAC and FC in all participants. Despite high nutrient density of the energy-adjusted diet of ageing people, the elderly consumed the lowest amounts of antioxidants and flavonoids due to the lowest food intake.

## 1. Introduction

Free radicals such as reactive oxygen species (ROS) and reactive nitrogen species (RNS) are defined as unstable and very reactive molecules having an unpaired electron in the outer orbit. They are generated by various endogenous systems in a healthy organism and play a beneficial role in maintaining homeostasis at the cellular level. However, an overproduction of free radicals causes the oxidation of biomolecules (proteins, amino acids, lipids, and DNA), which leads to cell injury and death. The removal of free radicals is achieved through enzymatic (e.g., superoxide dismutase, catalase, glutathione reductase, and glutathione peroxidase) and nonenzymatic (e.g., glutathione, arginine, taurine, creatine, zinc, selenium, vitamin C, vitamin E, vitamin A, and polyphenols) oxidative defense mechanisms. Prolonged exposure to free radicals and an inefficient antioxidant system may result in an oxidative stress [1]. The oxidative stress is considered to be involved in the pathophysiology of chronic diseases including cancer, atherosclerosis, diabetes, cardiovascular diseases, inflammation, stroke, and aging [2].

Flavonoids are the most common and the largest plant polyphenols that can prevent injury caused by free radicals [3, 4]. Flavonoids are divided into 6 different subclasses: flavan-3-ols (catechin, epicatechin), flavonols (quercetin kaempferol, myricetin), flavones (apigenin, luteolin), flavanones (hesperetin, naringenin), anthocyanins (cyanidin), and isoflavones (daidzein, genistein). They are present in significant amounts in commonly consumed beverages such as tea, coffee, and juices, fruits, vegetables, and grains. Prospective studies demonstrated that intake of flavonoids can be associated with a decreased risk of chronic diseases [5, 6]. Moreover, higher intake of flavonoids is associated with good health and wellbeing in older ages [7]. Most of the elderly, however, do not consume sufficient amounts of foods, what can predispose them to malnutrition and vulnerability to several stressors, including illness [8].

We hypothesize that the elderly people may not ingest adequate amounts of antioxidants and flavonoids. Therefore, the objective of this study was to determine sources and patterns of antioxidant and flavonoid intakes in the elderly (61–74 yrs) in comparison to the young (20–40 yrs) and middle age (41–60 yrs) groups in a cross-sectional study.

## 2. Materials and Methods

### 2.1. Study Population

Food consumption data were collected during the National Multicenter Health Survey (WOBASZ) study. The WOBASZ study was approved by the Bioethics Committee of the National Institute of Cardiology in Warsaw (number 708). This study was continued for 3 years (2003–2005) and included a representative sample of Polish adult population, randomly selected from over 26 million Polish inhabitants aged 20–74 years. The sampling method has been reported previously [9]. A sample of 19200 individuals was randomly chosen from the personal identification number (PESEL) database from each of the sixteen Polish provinces. Finally, 13545 people agreed to take part in the study, from which approx. 50% subjects complete a single 24-hour dietary recall questionnaire. The subjects (6661 adults: 3132 males and 3529 females) were divided into three age groups: (1) young adult: 20–40 years (1179 men, 1374 women); (2) middle age: 41–60 years (1375 men, 1513 women); (3) old age: 61–74 years (578 men, 642 women). Baseline characteristics of the participants are shown in Table 1.

### 2.2. Food Consumption

Daily food consumption was estimated by the single 24-hour dietary recall. Food portion sizes were estimated using an album of food photographs [13]. It was determined on this basis that 96 plant foods and beverages were consumed. All consumed items were grouped into 6 food categories: (1) beverages, (2) vegetables, (3) fruits and fruit jams, (4) cereal products, (5) chocolates, and (6) nuts and seeds.

### 2.3. Preparation of Food Samples

Food products were randomly purchased in triplicate at different local food markets. Edible raw parts of fruits, vegetables, and button mushrooms (approximately 200 g) were washed separately, sliced, and dried using an air-drier (MPM GP-101, Poland) at 60–70°C to dry matter for about 20 hours. Edible raw parts of pulses, nuts, seeds, bread, rolls, and cereal products (approximately 100 g) were dried at 120°C for 60 min in a convection air-oven (BMT, Czech Republic). Dried products were pulverized in a grinder and stored at room temperature in a desiccator in plastic containers until analysis.

Pulverized samples of fruits, vegetables, mushrooms, pulses, nuts, seeds, bread, rolls, cereal products, and raw samples of chocolates, jams, and processed tomato products were extracted according to Saura-Calixto and Gõni [14] and the procedure described previously [9].

Pulverized samples (0.25 g) of dried foods or 1 g of raw samples of chocolates, jams, and processed vegetables, dissolved in 5 mL of hot distilled water, were placed in test tubes with 10 mL of methanol/water solution (50 : 50, v/v) and the pH was adjusted to 2 using 2 M HCl. The mixture was vigorously shaken for 1 hour and then centrifuged. The resulting supernatants were collected. Then the residues were extracted with 10 mL of an acetone/water mixture (70/30, v/v) and the procedure was repeated. Both methanol and acetone extracts were combined and used for analyses. Tea, ground coffee, and hot cocoa infusions were prepared as follows: 1 g tea, ground coffee, or hot cocoa was extracted for 3 min with 100 mL of boiling distilled water. Drinking chocolate, soluble coffee (1 g) was dissolved in 100 mL of hot distilled water.

### 2.4. Flavonoid Assay

Flavonoid content in food samples was determined according to mostly applied spectrophotometric method based on the formation of aluminium-flavonoid complexes [9, 15].

Briefly, 1 mL of 2% aluminium trichloride (AlCl_3_) in methanol was mixed with the same volume of the extract. After 10 min of incubation at room temperature, spectral analysis was performed at 415 nm against a blank sample consisting of a 1 mL extract solution with 1 mL methanol without AlCl_3_. This method is selective mainly for flavonols and flavones (luteolin). Next, the procedure was repeated with NaNO_3_ and spectral analysis was performed at 510 nm. This method is selective for rutin, luteolin, and catechins [16]. The concentration of flavonoids in the samples was determined from the standard curve and expressed as quercetin equivalents (mg QE/100 g [mL] fresh mass).

### 2.5. FRAP Assay

The FRAP (ferric reducing antioxidant power) was determined with the Benzie and Strain method [17] according to the procedure described previously [9, 11, 12]. This method is based on the reduction of Fe^3+^-2,4,6-tripyridyl-s-triazine (TPTZ) complex to the TPTZ-Fe^2+^ form in the presence of antioxidants. The measurements were performed at 593 nm after 4 min incubation. The antioxidant potential of a sample was determined from the standard curve using FeSO_4_·7H_2_O and expressed briefly in mmol Fe^2+^/100 g [mL] and then as Trolox equivalents (mmol TE/100 g [mL] fresh mass). Most of the presented results, expressed as mmol Fe^2+^/100 g [mL], were previously published [11, 12].

### 2.6. Assessment of Flavonoid Intake and Antioxidant Capacity of Diet

Dietary flavonoid intake was estimated using daily food consumption data (based on the single 24-hour recall method) and the flavonoid content in foods consumed by the participants.

The own dietary database [11, 12] of the total antioxidant capacity of foods, determined using the FRAP assay, was used to calculate daily antioxidant capacity of the diet. FRAP database contains over 150 foods and food products, of which 96 plant foods were found to be consumed by the participants. Results presented in this database are comparable to other studies [10, 18]. Minor differences are due to some modifications of the FRAP method in different publications and a variety of food quality in different countries.

### 2.7. Statistical Analysis

The data analysis was performed using the Statistica 10.0 software (StatSoft, Inc.). The results were expressed as number, percentage, mean value, and standard deviation or 95% confidence interval. Normality of continuous data distribution was verified with the Shapiro-Wilk test. A comparison of the means of measurements was performed by one-way analysis of variance (ANOVA). Correlations between variables were calculated with the Pearson's test. Values of *p* < 0.05 were considered statistically significant.

## 3. Results

Baseline characteristics of the participants were presented in Table 1. Compared to the young group (20–40 year), the older groups (41–60 and 61–74 year) were characterized with higher BMI, total cholesterol, triglycerides, fasting glucose, homocysteine, and blood pressure. However, energy of the diet was the lowest in the elderly men and women.

The mean intake of foods in the diet of the studied population was shown in Table 2 [10]. Among beverages, the consumption of tea and coffee was predominant. Potatoes were the major vegetables, while apples were the most widely consumed fruits. In the group of cereal products the most popular was white bread. In comparison to the young (20–40 years) and the middle age (41–60 years) participants, the elderly men and women (61–74 years) consumed less beverages and vegetables. Cereals products, mainly white bread, were consumed in the largest amounts in men groups, especially young men (20–40 years). It is also worth noting that the elderly persons consumed the lowest amounts of chocolates among the studied groups. Although, minor intake of chocolates and nuts and seeds was showed for all studied population.

Total antioxidant capacity (TAC) [11, 12] and flavonoid content (FC) of the foods consumed by the participants were shown in Table 3. The studied foods were characterized by various TACs, which ranged between 0.02 mmol TE/100 g (in cucumbers) and 43.61 mmol TE/100 g (in walnuts). The highest TACs, exceeding 2 mmol TE/100 g/100 mL, were found in (given in descending order): walnuts, dark chocolate, sunflower seeds, semisweet chocolate, woodland strawberries, raspberries, bilberries and bilberry jam, hazelnuts, sour cherries, lingonberries, milk chocolate, fennel, northern cranberries, red wine, red cabbage, and strawberries.

The FC in the analyzed foods varied from 2.52 mg QE/100 g (in cucumbers) to 76.23 mg QE/100 g (in fennel). Fennel, sorrel, northern cranberries, red wine, walnuts, woodland strawberries, onions, bilberries, sour cherries, lingonberries, oranges, grapefruits, dark chocolate, sunflower seeds, plums, and strawberries were characterized by the highest flavonoid contents (over 30 mg QE/100 g [100 mL], given in descending order).

Table 4 illustrates the average and energy-adjusted TAC and FC in the diet (*per capita*) for both genders according to the age categories. The mean TAC of the diet was estimated to be 6041 *µ*mol TE/person/day and ranged from 5247 *µ*mol in the women aged 61–74 years to 6542 *µ*mol in the men aged 20–40 years. The mean FC in the diet was 276 mg QE/person/day and ranged from 238 mg in the women of 61–74 years to 304 mg in the men of 20–40 years. The TAC and FC in the diet were significantly the lowest for both genders aged 61–74 years in comparison to the young adult (20–40 years) and the middle-age (41–60 years) groups. It was primarily associated with the lowest consumption of foods in the elderly men and women. Nutrient density of the energy-adjusted diet showed that TAC and FC in the diet of elderly was similar or higher in comparison to younger groups of participants.

The major dietary contributors to the TAC and FC in all studied groups were: beverages (tea, coffee), fruits (apples, strawberries, plums, and sour cherries), and vegetables (potatoes, cabbage, and beetroots).

## 4. Discussion

This study attempts to establish dietary antioxidant capacity and flavonoid intake in the elderly Polish population. Antioxidant capacity of the diet was calculated using the own dietary database of the total antioxidant capacity of selected foods [11, 12], whereas flavonoid content in the diet was calculated after determining the flavonoids in foods consumed by the participants. To the best of our knowledge, this is the first attempt made to estimate the antioxidant and flavonoid intakes in the general Polish population in a cross-sectional study. In a previous study [19] we estimated dietary intake and patterns of polyphenol consumption in Polish adult population. Another Polish study [20] estimated intake of flavonoids in the diet of participants from the city of Krakow.

Elderly men and women were characterized with higher BMI, total cholesterol, triglycerides, fasting glucose, homocysteine, and blood pressure in comparison to the young participants, what can predispose this group to chronic diseases. Epidemiological studies have reported an inverse association between the risk of chronic diseases and the consumption of antioxidant-rich foods [21–23]. Total antioxidant capacity of a diet is the summation of antioxidant activities resulting not only from flavonoids but also from antioxidant vitamins C and E and carotenoids. Among them, flavonoids appear to be quantitatively the main dietary antioxidants [24].

The average dietary TAC in the own study (6041 *µ*mol TE/person/day) was comparable to that of the Mediterranean diet (6014 *µ*mol TE/person/day) [14]. In this study, however, despite increasing demand for antioxidants, mean dietary TAC was the lowest in the people aged 61–74 years, which usually suffer from several diseases. It is commonly known that food intakes in the elderly people are usually lower due to reduced appetite and thirst sensation [25]. In our study elderly consumed diminished amounts of foods with the lowest energy of the diet, what influenced antioxidant capacity of the whole diet. Despite high nutrient density of the energy-adjusted diet, elderly consumed lower antioxidants in comparison to younger groups. In this study contributions of tea (35%), coffee (20%), and apples (9%) to the intake of antioxidants were predominant. In the Spanish diet [14] coffee (45%) and wine (14%) were main contributors to the dietary TAC, whereas, in the Italian study it was coffee, wine, and fruit, which altogether described more than 50% of the total antioxidant intake [26]. Tea, dietary supplements, and fruits and fruit juices were the major sources of dietary TAC of the US population (28, 25, and 17%, resp.) [27]. Importantly, while aforementioned studies indicate that habitually consumed foods are sources of various amounts of antioxidants, the dietary recommendations for elderly people should encompass consumption of antioxidant rich foods locally available.

The mean estimated flavonoids intake in our study was 276 mg QE/person/day. This result is lower than the flavonoid intakes in Spanish (313 mg/person/day) [28] and the Australian studies (454 mg/person/day) [29], nevertheless higher than the intakes of flavonoid intake in Greek [30] and US [31] population (119 and 190 mg/person/day, resp.). These differences can be affected to some extent by various measurement methods. Moreover, flavonoid contents in foods are variable. Their content is dependent on several factors, such as ripeness at the time of harvest, environmental factors (soil type, sun exposure, and rainfall), processing, and storage [32]. Estimation of the dietary flavonoid content of the studied population was based on the national database with consideration of real consumption of foods available in the local food markets and included a representative sample of Polish adult population.

The contribution of each specific food to the FC of the diet is dependent on the dietary habits as well as the flavonoid contents in foods consumed by the participants. Our findings demonstrate that dietary FC of the studied population has declined along with the age and was the lowest in the men and women aged 61–74 years. The reduced FC in elderly was mainly associated with a diminished consumption of foods. In the current study over 90% FC in the diet were provided by beverages, fruits, and vegetables. More than 40% of FC in the diet was provided by tea (22%), coffee (8%), and apples (12%). In the Spanish diet main sources of flavonoid intake were apples (23%), red wine (21%), unspecified fruit (13%), and oranges (9%) [28].

Although flavonoids demonstrate numerous health benefits, they can be limited by low bioavailability, which vary among different flavonoid classes and individual flavonoids. Most flavonoids are presented in foods as glycosides, and only flavan-3-ols exist as aglycones. Absorption at the gastric level is possible for some flavonoids, such as quercetin, but not for their glycosides. Most of the glycosides probably resist acid hydrolysis in the stomach and thus arrive intact in the small intestine. Only aglycones and some glucosides can be absorbed in the small intestine in native form, whereas glycosides linked to a rhamnose moiety are absorbed less efficiently from the intestine and before absorption must be hydrolyzed by intestinal enzymes or by colonic microflora. During the process of the absorption, flavonoids are conjugated by methylation, sulfation, and glucuronidation. Therefore, after the flavonoid intake, metabolites of flavonoids in the blood and target organs are found, which may differ from the native substances in terms of biological activity [33, 34]. Therefore, it is important to study the correlation between antioxidant capacity of the blood and markers of oxidative stress in elderly [35].

## 5. Conclusion

This study estimated volume and patterns of the total flavonoid intakes as well as dietary antioxidant capacity in the elderly subjects in comparison to the young adult and middle-age groups. It was established that the consumption of tea, coffee, and apples was associated with the largest contributions to the dietary antioxidant capacity and the flavonoid content in the studied groups irrespective of age and gender. Despite high nutrient density of the energy-adjusted diet of ageing people, the elderly consumed the lowest amounts of antioxidants and flavonoids due to the lowest food intake.

## Figures and Tables

**Table 1:** Baseline characteristics of the participants (mean ± standard deviation or %).

Characteristics	Men			Women		
	20–40 y.	41–60 y.	61–74 y.	20–40 y.	41–60 y.	61–74 y.
	Number					
	1179	1375	578	1374	1513	642
Age (y.)	29.5 ± 6.3	50.2 ± 5.3	67.1 ± 3.9	29.6 ± 6.1	49.9 ± 5.3	67.1 ± 3.9
BMI (body mass index)	25.2 ± 3.8	27.4 ± 4.7	27.8 ± 4.5	23.4 ± 4.5	27.6 ± 5.4	29.2 ± 5.8
Total cholesterol (mmol/L)	4.9 ± 1.1	5.7 ± 1.1	5.6 ± 1.1	4.8 ± 1.0	5.7 ± 1.2	5.9 ± 1.2
HDL (mmol/L)	1.4 ± 0.4	1.4 ± 0.5	1.3 ± 0.4	1.6 ± 0.4	1.5 ± 0.4	1.5 ± 0.4
Triglycerides (mmol/L)	1.5 ± 1.1	1.9 ± 1.7	1.6 ± 0.9	1.1 ± 0.7	1.4 ± 0.9	1.7 ± 1.4
Fasting glucose (mmol/L)	4.7 ± 0.8	5.3 ± 1.6	5.6 ± 2.0	4.5 ± 0.8	5.0 ± 1.4	5.6 ± 2.0
Homocysteine (*µ*mol/L)	9.7 ± 3.4	11.2 ± 5.2	12.5 ± 5.3	8.1 ± 2.5	9.5 ± 4.1	11.6 ± 4.3
Systolic Blood Pressure (mmHg)	130 ± 12	140 ± 19	148 ± 23	117 ± 12	133 ± 21	150 ± 24
Diastolic Blood Pressure (mmHg)	79 ± 10	87 ± 12	86 ± 12	76 ± 9	84 ± 12	88 ± 12
Smoking (%)	41.8	44.9	25.9	26.3	30.1	5.6
Physical activity (%)						
Low level	30.4	32.7	37.4	35.2	37.1	49.6
Middle level	20.0	13.6	9.2	21.7	15.9	6.6
High level	49.6	53.7	53.4	43.1	47.0	43.8
Commune type (%)						
<8th inhabitants	37.1	35.4	36.3	38.5	36.9	34.5
8–40th inhabitants	36.6	34.2	37.5	35.3	36.6	33.7
>40th inhabitants	26.3	30.4	26.2	26.2	26.5	31.8
Marital status (%)						
Married	52.7	84.0	87.7	68.0	81.3	54.3
Single	47.3	16.0	12.3	32.0	18.7	45.7
Level of education (%)						
Under middle	54.0	65.4	73.2	37.3	52.6	69.4
Middle	36.1	25.6	17.9	48.1	37.0	24.2
High	9.9	9.0	8.9	14.6	10.4	6.4
Household per capita income (%)						
Low	89.1	90.2	84.1	92.8	92.1	88.8
Middle	7.5	6.0	12.9	4.7	5.5	10.0
High	3.4	3.8	3.0	2.5	2.4	1.2
Energy of the diet (kcal)	2815 ± 1142	2362 ± 927	2011 ± 751	1794 ± 715	1694 ± 698	1505 ± 586

**Table 2:** Daily plant food and beverages intake in the study groups (g [mL] fresh edible mass/person/day) [10].

Plant foods and beverages	Men (*n* = 3132) – mean (95% CI)			Women (*n* = 3529) – mean (95% CI)		
	20–40 years (*n* = 1179)	41–60 years (*n* = 1375)	61–74 years (*n* = 578)	20–40 years (*n* = 1374)	41–60 years (*n* = 1513)	61–74 years (*n* = 642)
Beverages	682 (645–719)	686 (647–725)	611 (551–671)	584 (551–617)	565 (543–587)	509 (453–565)
Tea infusion^1^	388 (371–405)	398 (383–413)	426 (401–451)	330 (317–343)	337 (325–349)	377 (358–396)
Coffee infusion^2^	154 (143–165)	162 (152–172)	116 (104–128)	192 (182–202)	194 (185–203)	105 (94–116)
Others^3^	140 (73–183)	126 (70–178)	69 (29–137)	62 (28–118)	34 (15–78)	27 (15–64)
Vegetables	611 (577–645)	576 (547–605)	498 (471–525)	441 (411–471)	430 (401–459)	408 (385–431)
Potatoes	371 (353–389)	337 (322–352)	300 (280–320)	240 (227–253)	234 (223–245)	232 (217–247)
Others^4^	240 (143–365)	239 (122–298)	198 (133–267)	201 (112–287)	196 (108–311)	176 (95–281)
Fruits and jams	172 (146–198)	174 (151–197)	190 (161–219)	203 (181–225)	225 (201–249)	190 (165–215)
Apples	105 (96–114)	112 (103–121)	119 (104–134)	120 (112–128)	128 (120–136)	111 (100–122)
Others^5^	67 (34–123)	62 (43–145)	71 (39–155)	83 (51–178)	97 (62–191)	79 (44–177)
Cereal products	264 (221–307)	217 (188–246)	187 (165–209)	144 (123–165)	143 (112–174)	136 (105–167)
White bread	165 (156–174)	142 (125–159)	112 (103–121)	66 (62–70)	72 (68–76)	64 (59–69)
Others^6^	99 (44–161)	75 (39–149)	75 (47–138)	78 (51–131)	71 (42–155)	72 (47–143)
Chocolates^7^	5 (2–8)	2 (1–3)	1 (0.5–1.5)	4 (2–6)	3 (2–4)	1 (0.5–1.5)
Nuts and seeds^8^	2 (0–5)	1 (0–2.5)	1 (0–2.5)	2 (0–5)	1 (0–2.5)	1 (0–2.5)

CI: confidence interval; *n*: number. ^1^Tea infusion: black tea, green tea, red tea, rooibos tea, and white tea. ^2^Coffee infusion: soluble coffee, ground coffee. ^3^Others: beer, red and white wine, hot cocoa, drinking chocolate, and juices: apple, orange, blackcurrant, and lemon. ^4^Others: fennel, sorrel, parsley roots, chives, red and green pepper, leeks, celery roots, lettuce, radish, cauliflower, tomato paste, ketchup, green beans, beans, peas, button mushrooms, white cabbage, red cabbage, Chinese cabbage, tomatoes, cucumbers, carrot, onions, and beetroots. ^5^Others: oranges, grapefruits, mandarins, bananas, strawberries, plums, pears, grapes, sour cherries, sweet cherries, apricots, red currants, nectarines, peaches, kiwi fruits, watermelon, northern cranberries, bilberries, woodland strawberries, lingonberries, raspberries, and jams (orange, bilberry, plum, black currant, sour cherry, strawberry, apricot, peach, and pineapple). ^6^Others: white rolls, wheat flour, wholegrain bread, noodles, buckwheat groats, barley groats, extruded rye bread, extruded graham bread, oats, and rice. ^7^Chocolates: dark, semisweet, milk, and white. ^8^Nuts and seeds: walnuts, sunflower seeds, pistachios, hazelnuts, peanuts, and pumpkin seeds.

**Table 3:** Mean TAC (mmol TE/100 g [mL] fresh mass) [11, 12] and FC (mg QE/100 g [mL] fresh mass) in plant foods and beverages.

	TAC	FC
*Plant foods and beverages *		
Beverages		
Red wine	2.29 ± 0.31	48.31 ± 4.12
Blackcurrant juice	1.15 ± 0.05	21.54 ± 3.63
Soluble coffee infusion	1.11 ± 0.09	15.92 ± 2.31
White tea infusion	0.83 ± 0.15	16.83 ± 1.74
Green tea infusion	0.69 ± 0.12	16.81 ± 1.52
Lemon juice	0.64 ± 0.09	5.93 ± 0.31
Black tea infusion	0.61 ± 0.11	15.92 ± 1.13
Orange juice	0.52 ± 0.12	10.43 ± 2.32
Drinking chocolate	0.44 ± 0.05	3.81 ± 0.33
Ground coffee infusion	0.44 ± 0.06	13.62 ± 0.74
Rooibos tea infusion	0.35 ± 0.03	13.54 ± 1.41
Red tea infusion	0.27 ± 0.05	13.62 ± 1.32
White wine	0.20 ± 0.09	4.11 ± 0.42
Apple juice	0.19 ± 0.01	7.23 ± 1.13
Hot cocoa	0.19 ± 0.06	2.73 ± 0.22
Beer	0.17 ± 0.04	3.21 ± 0.74
Vegetables, processed tomato products, pulses, and button mushrooms		
Fennel	2.51 ± 0.61	76.23 ± 12.31
Red cabbage	2.23 ± 0.46	28.62 ± 2.74
Sorrel	1.50 ± 0.41	62.29 ± 7.37
Beetroots	1.02 ± 0.03	25.38 ± 2.21
Green pepper	0.60 ± 0.04	9.37 ± 1.92
Cauliflower	0.54 ± 0.03	7.01 ± 0.33
Tomato paste	0.42 ± 0.04	16.09 ± 3.23
Radish	0.34 ± 0.06	8.79 ± 1.19
Peas	0.30 ± 0.06	8.91 ± 0.42
Green beans	0.27 ± 0.05	13.28 ± 1.51
Beans	0.26 ± 0.09	8.11 ± 0.76
White onions	0.23 ± 0.04	41.81 ± 3.17
White cabbage	0.22 ± 0.05	9.89 ± 1.12
Red pepper	0.21 ± 0.06	10.13 ± 1.58
Button mushrooms	0.20 ± 0.05	2.95 ± 0.31
Chinese cabbage	0.19 ± 0.03	9.79 ± 0.89
Parsley roots	0.16 ± 0.02	22.29 ± 2.88
Chives	0.16 ± 0.01	17.11 ± 3.12
Tomatoes	0.15 ± 0.04	12.19 ± 2.08
Carrots	0.15 ± 0.05	8.79 ± 1.26
Ketchup	0.15 ± 0.06	11.91 ± 2.56
Leeks	0.12 ± 0.02	8.82 ± 2.53
Celery roots	0.10 ± 0.02	8.89 ± 1.51
Lettuce	0.09 ± 0.01	6.78 ± 1.42
Potatoes	0.06 ± 0.01	3.91 ± 0.43
Cucumbers	0.02 ± 0.01	2.52 ± 0.19
Nuts and seeds		
Walnuts	43.61 ± 13.17	43.21 ± 9.72
Sunflower seeds	9.52 ± 0.37	31.29 ± 7.38
Hazelnuts	3.50 ± 1.03	18.69 ± 1.51
Pistachios	1.31 ± 0.80	14.19 ± 2.71
Peanuts	1.18 ± 0.09	12.51 ± 2.12
Pumpkin seeds	0.66 ± 0.05	8.63 ± 0.49

*Plant foods and food products *		
Fruits		
Woodland strawberries	7.71 ± 3.02	42.29 ± 5.21
Raspberries	6.87 ± 1.42	38.67 ± 2.82
Bilberries	5.92 ± 0.23	41.38 ± 6.43
Sour cherries	3.29 ± 0.19	37.49 ± 4.09
Lingonberries	3.10 ± 0.22	35.12 ± 3.76
Northern cranberries	2.45 ± 0.38	55.39 ± 6.88
Strawberries	2.21 ± 0.81	30.01 ± 3.94
Plums	1.12 ± 0.41	30.82 ± 3.08
Green grapes	1.08 ± 0.11	19.91 ± 2.19
Red currants	1.02 ± 0.09	18.42 ± 1.93
Grapefruits	0.94 ± 0.13	32.18 ± 4.77
Oranges	0.92 ± 0.19	33.11 ± 4.22
Kiwi fruits	0.69 ± 0.14	10.53 ± 0.79
Mandarins	0.68 ± 0.06	17.12 ± 2.68
Sweet cherries	0.66 ± 0.12	16.44 ± 2.48
Apricots	0.63 ± 0.17	13.81 ± 2.89
Nectarines	0.56 ± 0.39	12.63 ± 2.08
Peaches	0.55 ± 0.21	11.22 ± 1.36
Apples	0.44 ± 0.05	28.87 ± 3.37
Bananas	0.34 ± 0.05	12.12 ± 1.13
Watermelon	0.25 ± 0.01	5.19 ± 0.21
Pears	0.24 ± 0.01	15.81 ± 2.48
Fruit jams		
Bilberry jam	3.53 ± 1.55	12.19 ± 1.71
Blackcurrant jam	1.51 ± 0.19	10.88 ± 1.22
Strawberry jam	1.01 ± 0.49	8.77 ± 0.91
Plum jam	0.86 ± 0.18	10.56 ± 1.93
Sour cherry jam	0.86 ± 0.39	7.41 ± 1.29
Orange jam	0.27 ± 0.02	13.78 ± 2.12
Pineapple jam	0.21 ± 0.01	2.89 ± 0.54
Apricot jam	0.21 ± 0.06	4.58 ± 0.81
Peach jam	0.13 ± 0.04	2.63 ± 0.69
Bread, rolls and cereal products		
Buckwheat groats	1.33 ± 0.25	13.58 ± 1.21
Barley groats	0.62 ± 0.23	6.59 ± 0.52
Extruded rye bread	0.30 ± 0.21	6.31 ± 1.13
Extruded graham bread	0.28 ± 0.15	6.19 ± 0.88
Wholegrain bread (rye)	0.24 ± 0.05	8.81 ± 1.82
Wheat rolls	0.18 ± 0.01	6.09 ± 0.61
Oats	0.17 ± 0.05	4.77 ± 0.89
White bread (wheat)	0.17 ± 0.07	6.21 ± 0.79
Noodles	0.10 ± 0.04	2.82 ± 0.33
Wheat flour	0.06 ± 0.01	2.89 ± 0.19
Rice	0.05 ± 0.01	3.12 ± 0.47
Chocolates		
Dark chocolate	11.44 ± 1.26	31.31 ± 3.68
Semisweet chocolate	9.23 ± 1.47	26.12 ± 2.94
Milk chocolate	2.96 ± 0.32	11.18 ± 1.76
White chocolate	0.43 ± 0.04	8.21 ± 0.49

Data are expressed as mean ± standard deviation (*n* = 3); TE: Trolox equivalents; QE: quercetin equivalents; TAC: total antioxidant capacity; FC: flavonoid content.

**Table 4:** Mean and energy-adjusted antioxidant and flavonoid intake.

Plant foods and beverages	Men (*n* = 3132)			Women (*n* = 3529)		
	20–40 years (*n* = 1179)	41–60 years (*n* = 1375)	61–74 years (*n* = 578)	20–40 years (*n* = 1374)	41–60 years (*n* = 1513)	61–74 years (*n* = 642)
Mean antioxidant capacity [*µ*mol TE/person/day (95% CI)]	6542 (6120–6964)	6395 (5968–6822)	5852 (5737–5967)	5976 (5693–6259)	6233 (5983–6483)	5247 (4945–5549)

Energy-adjusted antioxidant intake [*µ*mol TE per 1000 kcal of the diet (95% CI)]	2324 (2174–2474)	2707 (2527–2888)	2910 (2853–2967)	3331 (3173–3489)	3679 (3532–3827)	3486 (3286–3687)

Main food contributors (% contribution to antioxidant content)	Beverages (58): tea (35), coffee (20); vegetables (10): potatoes (3), cabbage (2), and beetroots (2); fruits and jams (21): apples (9), strawberries (4), plums (2), and sour cherries (2); cereal products (5): white bread (3); chocolates (2); nuts and seeds (4)					

Mean flavonoid content [mg QE/person/day (95% CI)]	304 (291–317)	291 (279–311)	268 (256–280)	278 (266–290)	275 (264–286)	238 (227–249)

Energy-adjusted flavonoid intake [mg QE per 1000 kcal of the diet (95% CI)]	108 (103–113)	123 (118–132)	133 (127–139)	155 (148–162)	162 (156–169)	158 (151–165)

Main food contributors (% contribution to flavonoid content)	Beverages (47): tea (22), coffee (8); vegetables (18): potatoes (4), cabbage (3), onion (3), and beetroots (2); fruits and jams (27): apples (12); cereal products (7): white bread (3); chocolates (0.5); nuts and seeds (0.5)					

CI: confidence interval; *n*: number; TE: Trolox equivalents; QE: quercetin equivalents.
